# Impact of meropenem on *Klebsiella pneumoniae* metabolism

**DOI:** 10.1371/journal.pone.0207478

**Published:** 2018-11-15

**Authors:** Claudio Foschi, Melissa Salvo, Luca Laghi, Chenglin Zhu, Simone Ambretti, Antonella Marangoni, Maria Carla Re

**Affiliations:** 1 Microbiology, DIMES, University of Bologna, Bologna, Italy; 2 Centre of Foodomics, Department of Agro-Food Science and Technology, University of Bologna, P.za Goidanich, Cesena, Italy; 3 Microbiology Unit, S.Orsola-Malpighi Hospital, Bologna, Italy; Illinois Institute of Technology Paul V Galvin Library, UNITED STATES

## Abstract

The aim of this study was to analyze the metabolome of several *Klebsiella pneumoniae* strains characterized by different resistance patterns. A total of 59 bacterial strains (27 carbapenemase-negative and 32 carbapenemase-positive) were included and their metabolic features were assessed in basal conditions. Moreover, 8 isolates (4 wild-type and 4 KPC-producers) were randomly selected to evaluate the impact of sub-lethal concentrations of meropenem on bacterial metabolism. The metabolomic analysis was performed by ^1^H-NMR spectroscopy both on filtered supernatants and cell lysates. A total of 40 and 20 molecules were quantified in the intracellular and the extracellular metabolome, respectively. While in basal conditions only five metabolites showed significant differences between carbapenemase-positive and negative strains, the use of meropenem had a profound impact on the whole bacterial metabolism. In the intracellular compartment, a reduction of different overflow metabolites and organic acids (e.g. formate, acetate, isobutyrate) was noticed, whereas, in the extracellular metabolome, the levels of several organic acids (e.g. succinate, acetate, formate, lactate) and amino acids (aspartate, threonine, lysine, alanine) were modified by meropenem stimulation. Interestingly, carbapenemase-positive and negative strains reacted differently to meropenem in terms of number and type of perturbed metabolites. In wild-type strains, meropenem had great impact on the metabolic pathways related to methane metabolism and alanine, aspartate and glutamate metabolism, whereas in KPC-producers the effect was predominant on pyruvate metabolism. The knowledge about the bacterial metabolic profiles could help to set up innovative diagnostic methods and new antimicrobial strategies to fight the global crisis against carbapenemase-positive *K*. *pneumoniae*.

## Introduction

The global spread of carbapenemase-producing *Enterobacteriaceae* (CPE) is of great concern to health services worldwide [1, 2]. In particular, multi-drug resistant *Klebsiella pneumoniae* strains, harboring KPC enzymes, have been causing epidemics of international proportions [3, 4]. Healthcare-associated infections caused by CPE represent an alarming and dramatic problem for different reasons. Firstly, most carbapenemase-encoding genes are located on transferable genetic elements that are often associated with other antibiotic resistance genes, thus leading to their rapid transfer and to the spread of multi-drug resistant superbugs [5].

Moreover, the morbidity associated to CPE infections is usually high, with a relevant clinical and economic impact. Indeed, the mortality rate due to CPE infections is usually about 20–30% and can reach 70% in case of bacteremia or pneumonia in critically ill patients [6].

Finally, the therapeutic options for CPE infections are few and limited to old and toxic drugs, thus leading to the onset and spread of new resistance mechanisms (e.g. colistin resistance) [7].

In the last years, several approaches have been proposed to fight the global burden of CPE. On the one hand, screening and surveillance hospital protocols, as well as strict infection control measures (e.g. hand hygiene, patient isolation, personal protection equipment), have been adopted. On the other hand, rapid laboratory assays for the identification of CPE and new antimicrobials acting against KPC-producing *Enterobacteriaceae* (e.g. ceftazidime/avibactam) have been introduced in the diagnostic and clinical practice, respectively [8–12].

Nevertheless, the global crisis against multi-drug resistant CPE is still ongoing and constitutes a major public health challenge [13, 14]. For this reason, rapid and reliable diagnostic approaches, as well as new antimicrobial drugs, are urgently needed for a better management of CPE infections.

In this context, information about the metabolic profiles of CPE in basal conditions and under ‘antibiotic stress’ could represent an intriguing approach to obtain useful information to set up new diagnostic methods and to develop new antimicrobial strategies.

Proton-based nuclear magnetic resonance (^1^H-NMR) spectroscopy proved to be a fast and reliable analytical technique to detect and quantify low molecular weight metabolites in different matrices and biological fluids [15–17]. Moreover, it has been previously used to study the intracellular and extracellular metabolic profiles of different Gram-positive (e.g. lactobacilli and *Staphylococcus aureus*) and Gram-negative bacteria (e.g. *Acinetobacter baumannii*, *Pseudomonas aeruginosa*) [18–21].

It has been shown that data about the bacterial metabolome can be useful to get new insights on the microbial physiology and pathogenesis, to find correlations with the taxonomy and to distinguish bacterial strains on the basis of different growth patterns (e.g. planktonic vs biofilm) or based on various antimicrobial resistance phenotypes (e.g. methicillin-resistant vs methicillin-susceptible *Staphylococcus aureus*) [18–23].

The aim of this study was to characterize and compare the metabolic profiles of several carbapenemase-positive and carbapenemase-negative *K*. *pneumoniae* strains, by means of ^1^H-NMR spectroscopy, both in basal conditions and after the exposure to sub-lethal concentrations of meropenem. Major potential applications of this work concern the identification of specific metabolic biomarkers able to differentiate *K*. *pneumoniae* strains with different resistance patterns and the detection of critical metabolic pathways, to be used as potential targets of novel antimicrobial drugs.

## Materials and methods

### Bacterial strains

*K*. *pneumoniae* strains were isolated from surveillance and clinical samples submitted to the Microbiology Unit of S. Orsola-Malpighi University Hospital of Bologna (Italy) for routine diagnostic procedures.

The bacterial identification at the species level was obtained by means of a matrix-assisted laser desorption/ionization time-of-flight mass spectrometry (MALDI-TOF MS), using the VITEK MS system (bioMérieux), whereas antimicrobial susceptibility testing (AST) was performed using Vitek AST-N 201 cards on a Vitek 2 automated instrument (bioMérieux).

EUCAST criteria (www.eucast.org) were used for the interpretation of results and for the definition of antimicrobial susceptibility category. In the case of a strain resistant to a third-generation cephalosporins (i.e. cefotaxime and/or ceftazidime), the production of extended-spectrum β-lactamase enzymes was confirmed by double-disk synergy test [24]. Moreover, in the case of a minimum inhibitory concentration (MIC) of meropenem > 0.25 mg/L, a combination disk testing (disks of meropenem ± various inhibitors) was performed, in order to confirm the positivity for carbapenemase and to define the type of enzyme produced [25]. Temocillin susceptibility testing was used for the detection of OXA-48-like enzymes [26].

Starting from overnight grown bacterial colonies, the presence of a carbapenemase was further investigated by a genotypic method, based on the real-time amplification of the major carbapenemase genes (*bla*_KPC_, *bla*_IMP_, *bla*_VIM_, *bla*_NDM_ and *bla*_OXA-48_), using a commercially available assay (Xpert Carba-R, Cepheid).

For all the isolates, the exact MIC values of meropenem were calculated by means of a broth micro-dilution assay, following CLSI guidelines [27].

### Sample preparation

For each strain, bacteria were grown overnight in Mueller-Hinton (MH; Sigma-Aldrich) agar at 37°C in aerobic atmosphere. Afterwards, 400 μl of a 2 McFarland (McF) turbidity suspension (corresponding to 0.5 optical density [OD] at 550 nm) were inoculated into 5 mL of MH broth and incubated under shaking for 6–8 hours at 37°C. Each bacterial suspension was then adjusted to the same turbidity value (2.8 McF, corresponding approximately to 8 × 10^8^ colony forming units/mL and 0.7 OD at 550 nm) by adding MH broth. One mL of the bacterial suspension was centrifuged at 5,000 × g for 20 minutes at 4°C in order to separate the cell pellet from the supernatant. Then, supernatants were filtered through a 0.2 μm membrane filter to obtain cell free supernatants and stored at -80°C.

Cell pellets were then washed twice in sterile saline and internal metabolites were extracted with cold methanol, following the protocol described by Park *et al*., with slight modifications [28]. Briefly, the biomass pellets were suspended in 250 μL of 100% cold methanol (−20°C) and 250 μL of sterile saline, thoroughly vortexed and frozen at −80°C for 30 minutes. Afterwards, the cell pellets were allowed to thaw on ice for 30 minutes. The freeze–thaw cycles were repeated three times to cause the metabolites to leak from the cells. The suspensions were then centrifuged at 13,000 × g for 10 min. The supernatants were collected and stored on dry ice, whereas 500-μL aliquots of 100% cold methanol (250 μL) and sterile saline (250 μL) were added to the pellets. The entire above procedure was repeated, then the two aliquots were combined. Finally, the solutions were evaporated under reduced pressure and the samples were stored at −80°C.

At the time of metabolomic analysis, all the samples were gently thawed. Both the filtered supernatants and the cell lysates were analyzed by ^1^H-NMR to examine the extracellular and intracellular metabolome, respectively, as described below.

The same protocol was used to investigate the metabolic profile of 8 randomly selected *K*. *pneumoniae* strains (4 wild-type isolates and 4 KPC-producers) under ‘antibiotic-stress’. In particular, bacteria were allowed to grow for 6–8 hours in antibiotic-free MH broth and, in parallel, in MH broth added from the beginning of the incubation with sub-lethal concentrations (1/8 of the MIC value) of meropenem (Sigma-Aldrich).

As preliminary investigation, an evaluation of the bacterial growth curves in absence and in presence of various sub-MIC levels of meropenem (1/2, 1/4 and 1/8 of the MIC values) was performed by the measurement of the optical density values at different time points, at 550 nm. For a value of 1/8 of the MIC, the bacterial growth in presence of meropenem was comparable to the cultures without antibiotic (S1 Fig). Therefore, 1/8 of the MIC was chosen for antibiotic exposure assays, as the highest sub-lethal dose of meropenem that did not affect the bacterial growth.

After a sample size calculation based on previously published data [29], the 8 strains used for antibiotic-exposure experiments were selected randomly, considering that the metabolic profiles of the 59 *K*. *pneumoniae* isolates in basal conditions were very similar to each other (see Results section). To assess whether the randomly selected strains were representative of the corresponding groups, a rPCA model was calculated on all the strains and all the molecules of the extracellular compartment (S2 Fig). A screen test allowed to observe that three components offered the best compromise between variance explained and model complexity [30]. Along each of the three components, representing as much as the 74.8% of the total variance, the samples selected never exceeded 1.5 times the interquartile range of the corresponding group and could be therefore considered representative of the corresponding groups [31].

### Metabolomic analysis

The metabolomic investigation was performed by means of ^1^H-NMR spectroscopy, starting from the filtered supernatants (‘external metabolome’) and the cell lysates (‘internal metabolome’).

For each *K*. *pneumoniae* strain, 700 μl of cell-free supernatant and 100 μl of cellular lysate were added to 100 μl of a D_2_O solution of 3-(trimethylsilyl)-propionic-2,2,3,3-d4 acid sodium salt (TSP) 10 mM set to pH 7.0 ± 0.02 by means of a 1M phosphate buffer, containing also NaN_3_ 2 mM to avoid bacteria proliferation. Cellular lysate samples were also added with 600 μl of deionized water, to meet the sample volume specifications of the NMR probe.

^1^H-NMR spectra were recorded at 298 K with an AVANCE III spectrometer (Bruker, Milan, Italy) operating at a frequency of 600.13 MHz, equipped with Topspin software (Ver. 3.5) [17, 32]. The spectra were formed by 32K points, spanning a spectral width of 11.97 ppm, registered for 256 scans. Each spectrum was then processed (alignment, baseline adjustment) following the procedure previously described [17, 33, 34].

The signals were assigned by comparing their chemical shift and multiplicity with the Human Metabolome Database [35] and the compounds library (Ver. 10) of Chenomx software (Chenomx Inc., Canada, Ver. 8.3). Quantification of the molecules was performed in the first sample acquired by employing the added TSP as an internal standard. In the case of cell-free supernatant, the first sample was represented by the growth medium. In order to compensate for differences in solids content, any other sample was then normalized to such sample by means of probabilistic quotient normalization [36]. Integration of the signals was performed for each molecule by means of rectangular integration.

### Metabolic pathway analysis

MetaboAnalyst 3.0 (www.metaboanalyst.ca), a web server designed to permit comprehensive metabolomic data analysis, was used to evaluate the impact of sub-lethal meropenem concentration on the bacterial metabolic pathways [37].

The program is based on several databases, including KEGG (http://www.genome.jp/kegg/) and the Human Metabolome Database (http://www.hmdb.ca/) and identifies the pathways that are mostly perturbed.

The final list of altered metabolites that were significantly influenced by drug treatment, was analyzed by Metabolomics Pathway Analysis (MetPA) software in MetaboAnalyst. The built-in prokaryote (*Escherichia coli* K-12 MG1655) pathway library was used as reference. The following pathways analysis algorithms were employed: the hypergeometric test for over-representation analysis and the relative-betweenness centrality for pathway topology analysis [38, 39].

The output of this program marks a metabolic pathway as significant if a significantly higher number of compounds involved in that pathway is present in the input list than would be expected by random chance. In the graphical outputs (metabolome view), each dot represents one metabolic pathway. The circle size and color shade of each circle are based on pathway impact value and *P*-value (red being the most significant), respectively [38, 39].

### Statistical analysis

All statistical analyses were performed by using R computational language (www.r-project.org) and GraphPad Prism version 5.02 for Windows (GraphPad software).

Differences in the extracellular/intracellular metabolome composition between bacterial groups were assessed by means of Kruskal-Wallis test, two-tailed paired or unpaired Wilcoxon test or t-test, where appropriate (univariate analysis).

A robust principal component analysis (rPCA) (multivariate analysis) [40] was also performed to obtain an overview of the differences between non-stimulated and meropenem-stressed bacterial groups, on the basis of the significant metabolites found by means of the univariate analysis. The scoreplot—the representation of the samples from the new point of view—helps to visually inspect the trends underlying the samples, while the loadingplot (barplot) evidences the molecules mainly driving the trends. Statistical significance was determined at *P* < 0.05.

## Results

### Bacterial strains

A total of 59 *K*. *pneumoniae* strains were included in the study. On the basis of AST and phenotypic test results, *K*. *pneumoniae* isolates were categorized in carbapenemase-positive (n = 32) and carbapenemase-negative strains (n = 27). In particular, carbapenemase-positive strains included 24 KPC-producers, 4 strains positive for metallo-β-lactamases (MBL; specifically 3 NDM and 1 VIM) and 4 isolates with OXA-48 enzymes. The class of carbapenemase detected by phenotypic tests (combination disk testing) always agreed with the genotypic detection (real-time PCR) of carbapenemase genes.

The group of carbapenemase-negative strains included 16 wild-type *K*. *pneumoniae* strains and 11 ESBL-producers.

No significant differences were noticed with respect to bacterial growth between the different groups of strains.

The full list of bacterial strains with the source of isolation, the type of enzyme detected and the exact MIC values of meropenem are detailed in S1 Table.

### Metabolomic analysis

Consistent with previous reports on similar matrices [18, 41], a total of 40 and 20 molecules were detected and quantified by ^1^H-NMR analysis in the extracellular and intracellular metabolome, respectively. These metabolites mainly belong to the families of amino acids, organic acids and alcohols (S2 Table).

In order to highlight significant differences in the metabolic profiles of *K*. *pneumoniae* strains, different sets of analyses have been carried out, as follows: (i) in basal conditions, a comparison between carbapenemase-positive (n = 32) and carbapenemase-negative strains (n = 27), as well as between the three groups of strains harboring different types of carbapenemase (24 KPC, 4 MBL and 4 OXA-48), and between wild-type (n = 16) and ESBL-producing strains (n = 11); (ii) an analysis of a group of 8 randomly selected strains in antibiotic-free conditions and after meropenem exposure (1/8 of the MIC values), irrespective of the carbapenemase production; (iii) a comparison between non-stimulated and antibiotic-stressed bacteria, stratified by the carbapenemase production. In particular, for theses latter analyses, 4 KPC-producers (Kp08, Kp12, Kp15, Kp53) and 4 wild-type strains (Kp17, Kp25, Kp26, Kp61) were considered.

The results of these three different sets of analyses are detailed in the following paragraphs.

### (i) Metabolome of *K*. *pneumoniae* in basal conditions

Globally, the intracellular and extracellular metabolic profiles of the 59 *K*. *pneumoniae* strains in basal conditions were rather similar. Indeed, in antibiotic-free conditions, we found 2 metabolites in the intracellular metabolome (betaine and isobutyrate) and 3 molecules in the external metabolome (phenylalanine, sarcosine, valine), respectively, whose concentration differed significantly between carbapenemase-positive and carbapenemase-negative *K*. *pneumoniae* strains (Table 1). Similarly, in basal conditions, only 4 molecules showed significantly different concentrations in the comparison between KPC, MBL and OXA-48 positive strains (Table 2). Finally, two metabolites of the intracellular compartment (glutamate and acetate) were found to be significantly different when the metabolic profiles of wild-type and ESBL-positive strains were compared.

**Table 1:** List of metabolites whose concentration (mmol/L; mean ± SD) differed significantly between carbapenemase-positive (+) and carbapenemase-negative (-) *K*. *pneumoniae* strains in basal conditions.

Metabolite	Carbapenemase +	Carbapenemase -	Localization	*P*
Betaine	0.0084 ± 0.0044	0.0053 ± 0.0041	intracellular	0.009
Isobutyrate	0.0026 ± 0.0009	0.0034 ± 0.0009	intracellular	0.005
Phenylalanine	2.056 ± 0.08	2.083 ± 0.11	extracellular	0.025
Sarcosine	0.028 ± 0.004	0.035 ± 0.023	extracellular	0.048
Valine	2.486 ± 0.26	2.667 ± 0.36	extracellular	0.047

**Table 2:** List of metabolites whose concentration (mmol/L; mean ± SD) differed significantly between KPC, MBL and OXA-48 *K*. *pneumoniae* strains in antibiotic-free conditions.

Metabolite	KPC	MBL	OXA-48	Localization	*P*
Formate	0.07 ± 0.04	0.13 ± 0.07	0.122 ± 0.06	intracellular	0.02
Glycine	0.003 ± 0.001	0.001 ± 0.0006	0.002 ± 0.001	intracellular	0.03
Lactate	0.48 ± 0.24	0.85 ± 0.15	0.65 ± 0.19	extracellular	0.01
Propionate	1.25 ± 0.51	1.81 ± 0.06	1.48 ± 0.37	extracellular	0.03

### (ii) Metabolome of *K*. *pneumoniae* strains under ‘meropenem stress’

A total of 9 and 22 metabolites in the internal and external metabolome (S3 and S4 Tables), respectively, showed significant variations in antibiotic-stressed bacteria compared to non-stimulated strains, thus indicating that meropenem had globally a profound impact on the bacterial metabolism.

In the intracellular compartment, the most significant changes regarded the reduction of different overflow metabolites and organic acids (e.g. formate, acetate, isobutyrate), with the exception of succinate, showing higher levels after meropenem exposure.

When the extracellular metabolome was analyzed, it was possible to highlight that the levels of several metabolites, mostly organic acids and amino acids, were modified by the meropenem stimulation. As examples, aspartate, threonine, lysine and 2-hydroxyisobutyrate were secreted in significantly larger amounts, whereas a lower production of urocanate, succinate, acetate, ethanol, propionate and alanine was detected. Moreover, the antibiotic stimulation led to a higher consumption of formate, lactate and maltose by the bacterial cells.

**Fig 1:**
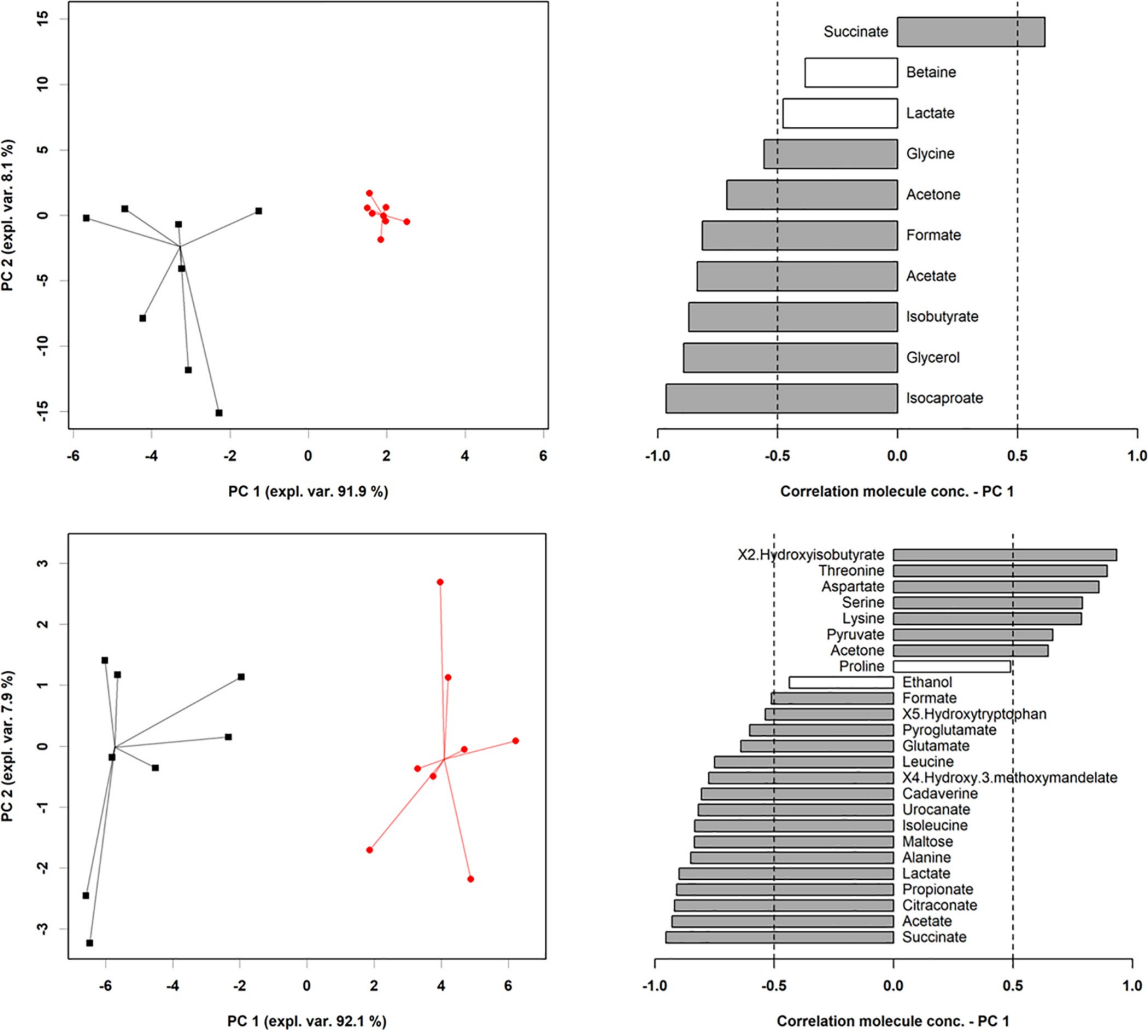
**rPCA model calculated on the space constituted by the concentration of the intracellular (upper panel) and extracellular (lower panel) molecules that significantly differed between non-stressed and meropenem-stressed bacterial strains.** In the scoreplot (left), non-stressed strains and meropenem-stressed strains are represented with black square and red dots respectively, with lines connecting each strain to the median of its group. In the barplot (right), describing the correlation between the concentration of each molecule and its importance over PC1, dark gray bars highlight statistically significant correlations (*P*<0.05). In the barplot, bars pointing to the left denote metabolites that were more abundant in non-stressed cells, while bars pointing to the right denote molecules that were more abundant in meropenem-stressed bacteria.

In order to investigate underlying trends in the metabolome subspace represented by these molecules, their concentrations were employed as the base for a rPCA model. In the intracellular metabolome, PC1, explaining the 91.9% of the total variance, effectively accounted for the stress connected to the presence of a meropenem, with non-stimulated strains appearing at low PC1 scores and clearly separated from antibiotic-stressed *K*. *pneumoniae* isolates (*P*<0.001) (Fig 1). The correlation between the concentration of each molecule and its importance over PC1 served as a handy way to rank the molecules in order of importance in determining such grouping. As pointed out in Fig 1, higher concentrations of succinate and lower concentrations of isocaproate and glycerol were the most significant changes after the stimulation with sub-lethal concentration of meropenem in the internal metabolome.

Similarly, in the external metabolome, non-stimulated strains were clearly separated from meropenem-stressed isolates along PC1, explaining the 92.1% of the total variance (Fig 1).

Higher concentrations of hydroxyisobutirate, threonine and aspartate and lower concentrations of succinate, acetate and citraconate were the main characteristics of the stimulated *K*. *pneumoniae* strains in contrast to non-stimulated bacteria (Fig 1).

Considering globally the data about the bacterial cell lysates and the supernatants, the analysis with MetaboAnalyst allowed to identify the specific metabolic pathways most affected by drug exposure.

**Fig 2:**
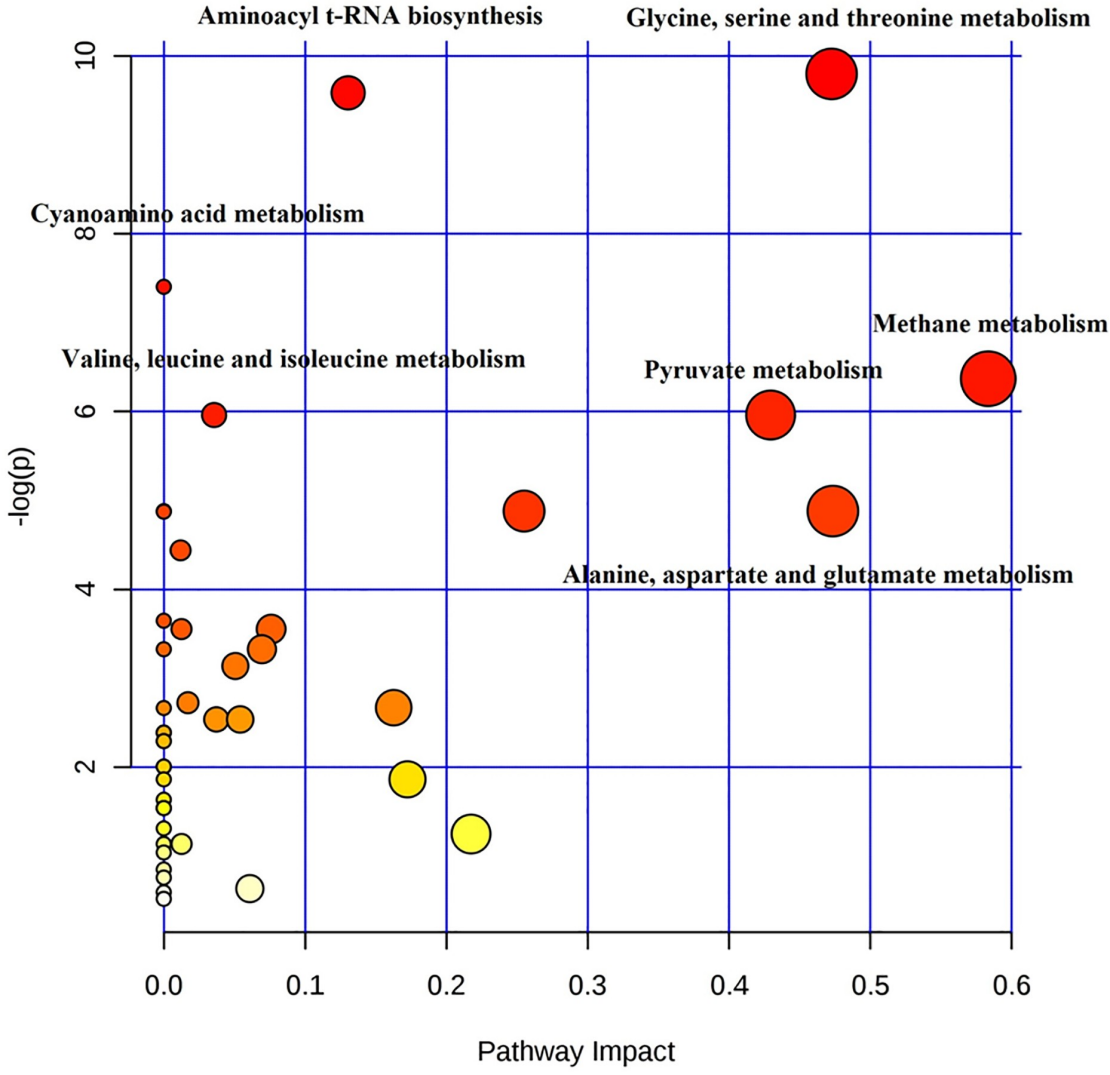
Metabolome view of the metabolic pathways altered after meropenem exposure in the group of the 8 selected *K*. *pneumoniae* strains. Each dot represents a unique metabolic pathway, with the dot size corresponding to the pathway impact score and the dot color (red being the most significant) corresponding to the–log(*P*) value.

As shown in Fig 2, the impact of meropenem on *K*. *pneumoniae* was dramatic: six metabolic pathways showed significant *P*-value following exposure (top portion with y-axis higher or equal to 5: glycine, serine and threonine metabolism; aminoacyl-tRNA biosynthesis; cyanoamino acid metabolism; methane metabolism; valine, leucine and isoleucine biosynthesis; pyruvate metabolism), and four metabolic pathways showed great pathway impact (x-axis larger or equal to 0.4: glycine, serine and threonine metabolism; methane metabolism; pyruvate metabolism; alanine, aspartate and glutamate metabolism).

### (iii) Analysis of the metabolic response to meropenem of wild-type and KPC-producing strains

As shown in Tables 3 and 4, the presence of sub-lethal concentration of meropenem led to different metabolic variations in the comparison between carbapenemase-negative (4 wild-type) and carbapenemase-positive strains (4 KPC-producers), in terms of both number and type of perturbed metabolites.

Indeed, in the group of wild type isolates, a total of 21 molecules was altered by the drug exposure, whereas 26 metabolites showed significant changes in the group of KPC-producers after meropenem stimulation. Among those molecules, 14 were altered in the two groups in the same direction (increase/reduction), while several others were perturbed only in one of the two groups (e.g. acetate in the intracellular metabolome of wild-type strains; urocanate and cadaverine in the external metabolome of KPC-positive isolates).

**Fig 3:**
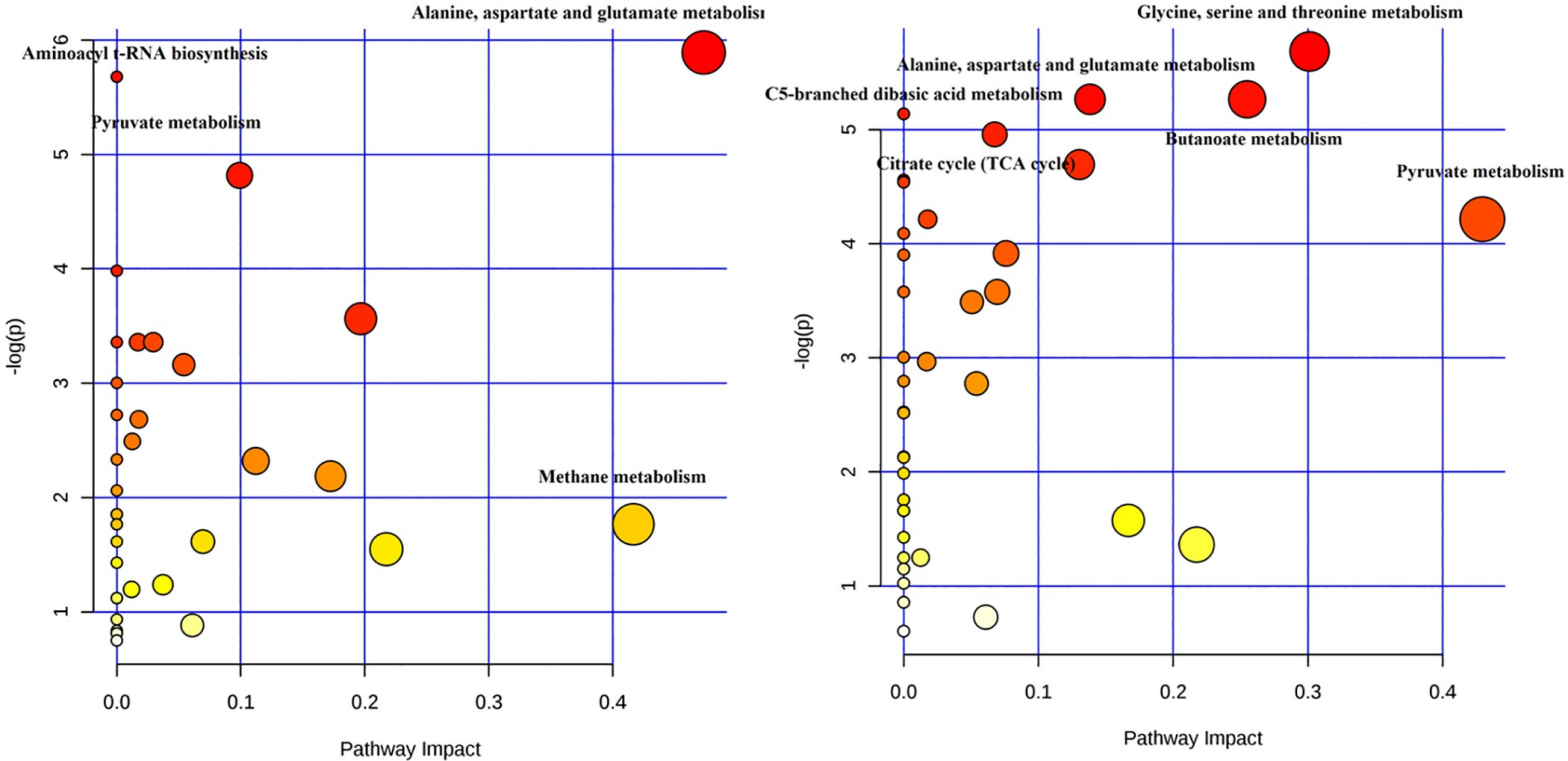
**Metabolome view of the metabolic pathways altered after meropenem exposure in the group of wild-type (left) and KPC-producing (right) *K*. *pneumoniae* strains.** Each dot represents a unique metabolic pathway, with the dot size corresponding to the pathway impact score and the dot color (red being the most significant) corresponding to the–log(*P*) value.

Considering the altered metabolites, an analysis of the most affected metabolic pathways was performed. In wild-type *K*. *pneumoniae* (Fig 3), three metabolic pathways were significantly altered after the drug exposure (alanine, aspartate and glutamate metabolism; aminoacyl-tRNA biosynthesis; pyruvate metabolism), and two showed great pathway impact (methane metabolism; alanine, aspartate and glutamate metabolism).

**Table 3:** List of intracellular/extracellular metabolites that significantly differed after the exposure with sub-lethal concentrations of meropenem in the group of carbapenemase-negative (wild-type) *K*. *pneumoniae* isolates. The concentration of metabolites is expressed as mmol/L (mean ± SD).

Metabolite	No stress	Meropenem stress	Localization	Variation	*P*
Formate	0.057 ± 0.02	0.022 ± 0.004	intracellular	↓	0.04
Glycerol	0.012 ± 0.003	0.006 ± 0.001	intracellular	↓	0.04
Lysine	0.024 ± 0.007	0.033 ± 0.009	intracellular	↑	0.025
Acetate	0.142 ± 0.02	0.023 ± 0.004	intracellular	↓	0.004
Isobutyrate	0.003 ± 0.001	0.0006 ± 0.0002	intracellular	↓	0.02
Isocaproate	0.008 ± 0.002	0.001 ± 0.0007	intracellular	↓	0.02
Citraconate	0.018 ± 0.001	0.014 ± 0.001	extracellular	↓	0.008
Maltose	0.025 ± 0.006	0.006 ± 0.002	extracellular	↓	0.006
Lactate	0.642 ± 0.199	-0.038 ± 0.006	extracellular	↓	0.006
Threonine	0.396 ± 0.633	1.915 ± 0.482	extracellular	↑	0.006
Agmatine	0.449 ± 0.015	0.349 ± 0.031	extracellular	↓	0.02
Lysine	0.815 ± 0.958	2.050 ± 0.197	extracellular	↑	0.04
Aspartate	0.678 ± 0.667	1.695 ± 0.119	extracellular	↑	0.04
Sarcosine	0.030 ± 0.001	0.025 ± 0.003	extracellular	↓	0.04
Succinate	5.015 ± 0.975	1.900 ± 0.218	extracellular	↓	0.01
Glutamate	12.70 ± 0.200	12.38 ± 0.206	extracellular	↓	0.04
Acetone	0.050 ± 0.001	0.055 ± 0.0006	extracellular	↑	0.003
Acetate	14.15 ± 1.872	8.815 ± 0.931	extracellular	↓	0.01
Alanine	3.878 ± 0.379	3.215 ± 0.097	extracellular	↓	0.03
2-Hydroxyisobutyrate	0.051 ± 0.023	0.193 ± 0.006	extracellular	↑	0.002
Propionate	1.545 ± 0.477	0.284 ± 0.225	extracellular	↓	0.005

**Table 4:** List of intracellular/extracellular metabolites that significantly differed after the exposure with sub-lethal concentrations of meropenem in the group of carbapenemase-positive (KPC) *K*. *pneumoniae* isolates. The concentration of metabolites is expressed as mmol/L (mean ± SD).

Metabolite	No stress	Meropenem stress	Localization	Variation	*P*
Lactate	0.025 ± 0.005	0.009 ± 0.006	intracellular	↓	0.03
Glycerol	0.015 ± 0.003	0.006 ± 0.001	intracellular	↓	0.002
Succinate	0.010 ± 0.002	0.025 ± 0.004	intracellular	↑	0.02
Acetone	0.002 ± 0.0007	0.001 ± 0.0005	intracellular	↓	0.03
Ethanol	0.005 ± 0.001	0.0004 ± 0.0005	intracellular	↓	0.008
Isobutyrate	0.003 ± 0.001	0.0009 ± 0.0004	intracellular	↓	0.005
Isocaproate	0.008 ± 0.001	0.0008 ± 0.0004	intracellular	↓	0.001
5-Hydroxytryptophan	0.941 ± 0.182	0.573 ± 0.170	extracellular	↓	0.04
Fumarate	0.295 ± 0.080	0.114 ± 0.018	extracellular	↓	0.03
Urocanate	0.016 ± 0.003	0.001 ± 0.001	extracellular	↓	0.002
Citraconate	0.018 ± 0.001	0.014 ± 0.001	extracellular	↓	0.0004
Maltose	0.014 ± 0.002	0.006 ± 0.001	extracellular	↓	0.03
Lactate	0.735 ± 0.235	-0.041 ± 0.005	extracellular	↓	0.007
Serine	0.386 ± 0.260	2.125 ± 0.241	extracellular	↑	0.005
4-H-3-methoxymandelate	0.070 ± 0.008	0.055 ± 0.004	extracellular	↓	0.03
Threonine	0.154 ± 0.152	2.685 ± 0.248	extracellular	↑	0.0005
Lysine	0.463 ± 0.205	2.170 ± 0.08	extracellular	↑	0.0001
Cadaverine	2.783 ± 0.349	0.968 ± 0.136	extracellular	↓	0.0016
Aspartate	0.396 ± 0.362	1.825 ± 0.092	extracellular	↑	0.0026
Pyroglutamate	1.170 ± 0.03	1.113 ± 0.047	extracellular	↓	0.01
Succinate	4.830 ± 1.15	1.793 ± 0.145	extracellular	↓	0.01
Pyruvate	0.139 ± 0.196	1.092 ± 0.194	extracellular	↑	0.008
Acetate	14.78 ± 0.95	6.583 ± 0.614	extracellular	↓	0.0004
Alanine	4.055 ± 0.369	3.190 ± 0.093	extracellular	↓	0.01
2-Hydroxyisobutyrate	0.042 ± 0.013	0.192 ± 0.007	extracellular	↑	0.0006
Propionate	1.618 ± 0.100	-0.020 ± 0.031	extracellular	↓	< 0.0001

Conversely, in KPC-producing strains (Fig 3), five metabolic pathways showed significant *P*-values following meropenem stimulation (glycine, serine and threonine metabolism; alanine, aspartate and glutamate metabolism; butanoate metabolism; C5-branched dibasic acid metabolism; citrate cycle or tricarboxylic acid cycle) and one showed great pathway impact (pyruvate metabolism).

## Discussion

To the best of our knowledge, this is the first report about the analysis of the metabolic features of several *K*. *pneumoniae* strains characterized by different resistance patterns before and after the exposure to meropenem. Previous works have investigated the volatile metabolome of *K*. *pneumoniae* in human blood or during in vitro growth, but none has examined the effect of meropenem on these bacteria, divided according to carbapenemase production [42–44].

At first, we looked for differences in the metabolic profiles between carbapenemase-positive and carbapenemase-negative *K*. *pneumoniae* strains in the absence of a meropenem exposure, as well as between the strains harboring different types of carbapenemase. In basal conditions, only few metabolites showed significant differences between these groups of isolates. This result agrees with a previous report about *S*. *aureus*, demonstrating that without exposure to antibiotics, the concentrations of only 5 metabolites varied significantly in comparison of isogenic pairs of methicillin-resistant (MRSA) and methicillin-susceptible (MSSA) strains [29].

Afterwards, we evaluated the global effect of sub-lethal concentrations of meropenem on the bacterial metabolome. To this purpose, we randomly selected 4 wild-type *K*. *pneumoniae* isolates and 4 strains positive for KPC enzymes, representing the commonest types of carbapenemase in many parts of the world [45].

Irrespective of the resistance pattern, the presence of meropenem induced profound perturbations in the bacterial metabolome. Indeed, the exposure to meropenem led to significant variations in the levels of different amino acids and organic acids with a great impact on several metabolic pathways including pyruvate metabolism and alanine, aspartate and glutamate metabolism.

It has been shown that β-lactams, besides their primary mechanism of action (i.e. inhibition of cell-wall synthesis), cause subsequent metabolic changes that occur downstream of the interaction of the antibiotic with their target, playing an important role in bacterial lethality [46, 47].

Effectively, β-lactams display a strong perturbations on bacterial metabolic profiles with the increase or decrease in the concentrations of several metabolites, suggesting that antibiotic treatment have broad and complex effects on metabolism and do not simply quench all metabolic activities [48].

In agreement with our findings, it has been previously shown, that the exposure to lethal or sub-lethal concentrations of β-lactams induces significant changes in metabolites involved in several pathways, like glycolysis, acetate metabolism, tricarboxylic acid (TCA) cycle, NAD and amino acids metabolism [48–50].

For example, in *S*. *aureus*, lethal doses of ampicillin induce a higher consumption of glucose, glutamine, valine, leucine and tyrosine, as well as a higher secretion of aspartate, glutamate, isobutyrate, acetate and pyruvate [49].

Similarly, in *Escherichia coli*, ampicillin leads to an increase of amino acids metabolites at 60 and 90 minutes post-treatment, as well as to higher concentrations of metabolites involved in central carbon metabolism, as citrate and succinate [48].

Comparing these data with our results, we noticed that some modifications showed similar trends (e.g. higher levels of pyruvate and aspartate), whereas others were opposed to those described for *E*. *coli* and *S*. *aureus* (e.g. variations of succinate and glutamate).

It should be considered that the bacterial metabolic response to an antibiotic is not only specific for each type of microorganism, but also depends on many other factors, as the type and dose (lethal/sub-lethal concentrations) of antimicrobial and the time of the analysis following the drug treatment.

Probably, the most interesting data concerned the assessment of the bacterial response to meropenem, stratified by the capability of carbapenemase production (wild-type strains vs KPC-producers).

It has been reported that KPC-producing *K*. *pneumoniae* strains differs from carbapenemase-negative due to the presence of the *bla*_KPC_ gene, carried on large plasmids and typically present in a Tn3-based transposon. This gene encodes for the KPC enzyme, responsible for high levels of penicillin and cephalosporin resistance and variable carbapenem resistance and it is usually accompanied by other drug resistance determinants (e.g. aminoglycosides, quinolones, trimethoprim and tetracyclines) [44, 51].

Hence, we hypothesized that even the exposure to sub-lethal levels of meropenem would cause different modifications in the metabolome between carbapenemase-negative and positive isolates.

Indeed, the bacterial response to meropenem differed significantly between wild-type and KPC-positive strains, in terms of type of altered metabolites and impaired metabolic pathways.

It has been previously shown that, after the exposure to sub-lethal doses of methicillin, MRSA and MSSA strains have different metabolic reactions, regarding the type, the number and the trend of change of metabolites, with great impact on different metabolic pathways. Globally, the analysis suggested that the metabolic activities of MSSA strains were more susceptible to the perturbation of methicillin exposure compared to the MRSA strains [29, 52].

Conversely, in this work, we noticed that carbapenemase-positive *K*. *pneumoniae* strains were not more resistant to the metabolic changes caused by meropenem compared to wild-type isolates. Rather, even though in presence of common metabolic alterations, some variations were specific for KPC-producers and others acted as biomarkers for wild-type isolates.

We are fully aware that some limitations could have affected our results. Among all, the relatively low sensitivity of ^1^H-NMR spectroscopy could have led to miss significant molecules in the metabolome of *K*. *pneumoniae* strains with a partial representation of the metabolic pathways perturbed by the meropenem stimulation. As an order of magnitude, ^1^H-NMR has been shown to detect metabolites with concentrations of at least 1 to 5 μM, higher than other metabolomics platforms (e.g. mass spectrometry) [53–55]. On the other hand, it is worth emphasizing some important strengths of the metabolomic analysis by ^1^H-NMR: (i) it is an intrinsically quantitative technique, (ii) the experimental protocol requires a very limited sample manipulation, allowing to process a large number of samples simultaneously [15, 56].

## Conclusions

In conclusion, the metabolomic analysis of the bacterial isolates by ^1^H-NMR allowed to detect and quantify a list of molecules that significantly differed between carbapenemase-positive and carbapenemase-negative strains, both in antibiotic-free conditions and under ‘meropenem stress’.

The data obtained could serve to set up innovative diagnostic strategies based on metabolic biomarkers, as well as to elucidate the action of β-lactams on the bacterial metabolome. The knowledge about these cellular responses are essential for improving existing therapies and identifying novel therapeutic approaches [46, 47]. In this way, it could be possible to open up significant perspectives for the control of the spread of CPE, still representing a worrisome emergency for public health structures and healthcare systems worldwide [57, 58].

Future perspectives will include the analysis of a larger panel of *K*. *pneumoniae* strains, evaluating the effect of different types of antimicrobials and various combinations of drugs on the bacterial metabolome. Moreover, the study of the effect of lethal concentrations of beta-lactams will allow the identification of the earliest and most abundant metabolites associated with the bacterial death. In this way, innovative approaches for the evaluation of the effectiveness of several antimicrobials against carbapenemase-positive *K*. *pneumoniae* could be possible.

Finally, it will be crucial to evaluate the metabolic profiles of *K*. *pneumoniae* strains getting deeper insights into their genetic background (e.g. sequence types by MLST or distinction between different classes of KPC enzymes), in order to understand if the observed data are limited to specific clones or, if they are common to bacterial isolates with different genetic origins.

## Supporting information

S1 Fig Bacterial growth curves at different sub-lethal levels of meropenem MIC. Panel A: carbapenemase-positive (KPC-producers) strains; Panel B: carbapenemase-negative (wt) strains. The growth was determined by the measurement of the optical density at 550 nm (OD_550_). For each time point, the mean value ± SEM (standard error of the mean) of the OD related to the strains selected for meropenem experiments is shown.
(TIF)

S2 Fig (A) Scoreplot of an rPCA model calculated on the space constituted by the concentration of the 40 molecules identified in the extracellular metabolome. Black (Carbapenemase-negative; C-) and red (Carbapenemase-positive; C+) lines connect each strain to the median of its group, while circles evidence the 8 samples considered to investigate the effect of meropenem. (B-D) The position of the samples along PC1, 2 and 3 is summarized as boxplots. The dashed lines evidence the inter-quartile distance multiplied by 1.5.
(TIFF)

S1 Table List of *Klebsiella pneumoniae* strains included in the study. wt: wild-type (no acquired resistance mechanisms); ESBL: extended-spectrum β-lactamase; MBL: metallo-β-lactamase; NDM: New Delhi metallo-β-lactamase; VIM: Verona Integron-encoded metallo-β-lactamase.
(DOCX)

S2 Table List of the molecules found in the intracellular and extracellular metabolome of *Klebsiella pneumoniae* strains. (DOCX)

S3 Table List of intracellular molecules that significantly differed after meropenem exposure in the group of selected *K*. *pneumoniae* isolates, irrespective of the carbapenemase production. The concentration of metabolites is expressed as mmol/L (mean ± SD).
(DOCX)

S4 Table List of extracellular molecules that significantly differed after meropenem exposure in the group of selected *K*. *pneumoniae* isolates, irrespective of their carbapenemase production. The concentration of metabolites is expressed as mmol/L (mean ± SD).
(DOCX)
