# Supplementary figures and images for: Impact of meropenem on Klebsiella pneumoniae metabolism
Source: PLoS One. 2018 Nov 15;13(11):e0207478. doi: 10.1371/journal.pone.0207478 (PMC6237392; doi:10.1371/journal.pone.0207478)

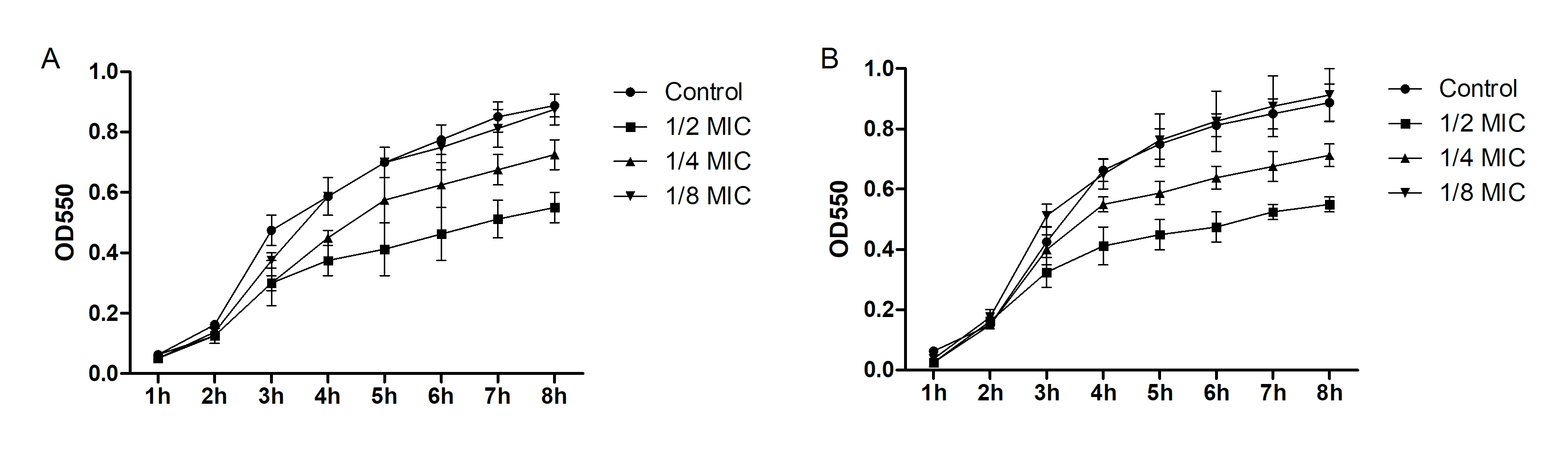

Supplement: S1 Fig — Panel A: carbapenemase-positive (KPC-producers) strains; Panel B: carbapenemase-negative (wt) strains. The growth was determined by the measurement of the optical density at 550 nm (OD550). For each time point, the mean value ± SEM (standard error of the mean) of the OD related to the strains selected for meropenem experiments is shown. (TIF) [file pone.0207478.s001.tif]

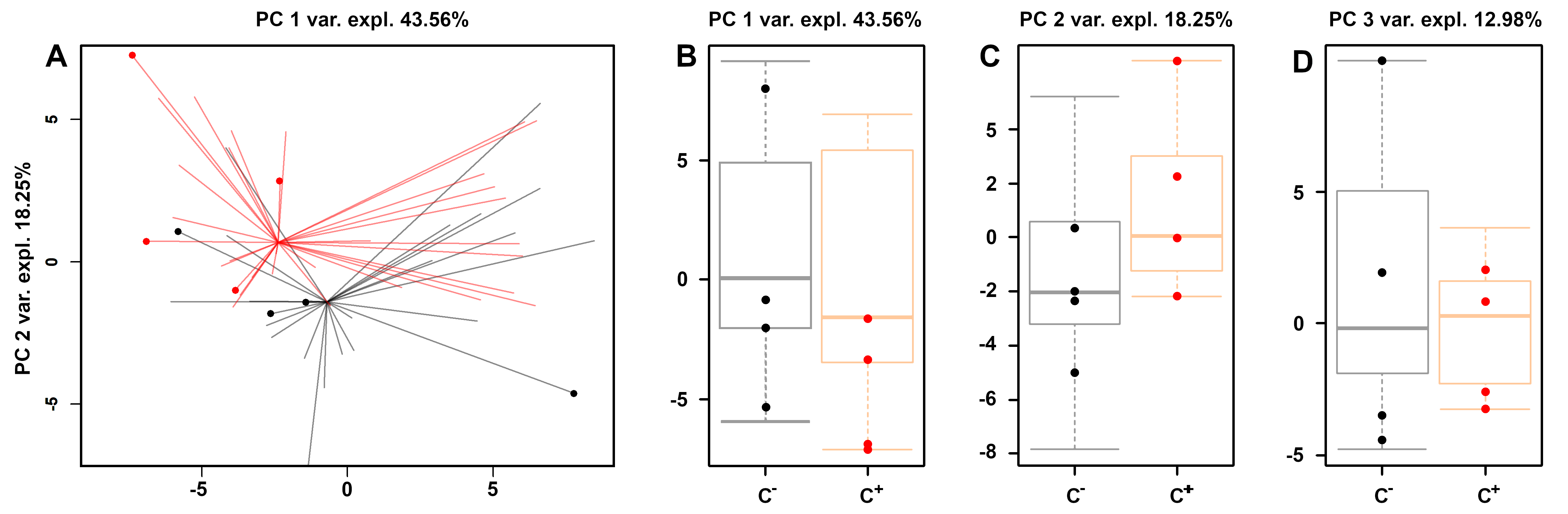

Supplement: S2 Fig — (A) Scoreplot of an rPCA model calculated on the space constituted by the concentration of the 40 molecules identified in the extracellular metabolome. Black (Carbapenemase-negative; C-) and red (Carbapenemase-positive; C+) lines connect each strain to the median of its group, while circles evidence the 8 samples considered to investigate the effect of meropenem. (B-D) The position of the samples along PC1, 2 and 3 is summarized as boxplots. The dashed lines evidence the inter-quartile distance multiplied by 1.5. (TIFF) [file pone.0207478.s002.tiff]
