# Supplementary material for: Impact of meropenem on Klebsiella pneumoniae metabolism
Source: PLoS One. 2018 Nov 15;13(11):e0207478. doi: 10.1371/journal.pone.0207478 (PMC6237392; doi:10.1371/journal.pone.0207478)
Supplement: S1 Table — wt: wild-type (no acquired resistance mechanisms); ESBL: extended-spectrum β-lactamase; MBL: metallo-β-lactamase; NDM: New Delhi metallo-β-lactamase; VIM: Verona Integron-encoded metallo-β-lactamase. (DOCX) [file pone.0207478.s003.docx]

| **Strains** | **Source of isolation** | **Carbapenemase**  **production** | **Resistance**  **mechanism** | **MIC Meropenem**  **(mg/L)** |
| --- | --- | --- | --- | --- |
| Kp01 | urine | - | ESBL | 0.03 |
| Kp02 | urine | - | ESBL | 0.03 |
| Kp03 | rectal swab | + | MBL (NDM) | 16 |
| Kp04 | rectal swab | + | MBL (NDM) | 32 |
| Kp05 | rectal swab | + | OXA-48 | 0.5 |
| Kp06 | blood | - | ESBL | 0.03 |
| Kp07 | rectal swab | + | OXA-48 | 8 |
| Kp08 | blood | + | KPC | 64 |
| Kp09 | blood | + | KPC | 512 |
| Kp10 | blood | + | KPC | 4 |
| Kp12 | blood | + | KPC | 256 |
| Kp13 | blood | + | KPC | 16 |
| Kp14 | blood | + | KPC | 32 |
| Kp15 | blood | + | KPC | 512 |
| Kp16 | blood | + | KPC | 256 |
| Kp17 | urine | - | wt | 0.06 |
| Kp18 | urine | - | wt | 0.015 |
| Kp20 | urine | - | wt | 0.015 |
| Kp21 | urine | - | wt | 0.015 |
| Kp23 | urine | - | wt | < 0.004 |
| Kp24 | urine | - | wt | < 0.004 |
| Kp25 | urine | - | wt | 0.125 |
| Kp26 | wound swab | - | wt | 0.06 |
| Kp27 | urine | - | ESBL | 0.008 |
| Kp29 | rectal swab | - | ESBL | 0.125 |
| Kp30 | genital swab | - | wt | 0.008 |
| Kp31 | genital swab | - | wt | 0.015 |
| Kp32 | wound swab | - | ESBL | < 0.004 |
| Kp33 | wound swab | - | ESBL | 0.015 |
| Kp34 | sputum | - | wt | 0.015 |
| Kp35 | biopsy | - | ESBL | 0.06 |
| Kp36 | rectal swab | + | OXA-48 | 1 |
| Kp37 | urine | - | ESBL | 0.125 |
| Kp38 | rectal swab | + | MBL (NDM) | 16 |
| Kp39 | rectal swab | + | KPC | 8 |
| Kp40 | rectal swab | + | MBL (VIM) | 16 |
| Kp41 | rectal swab | + | KPC | 32 |
| Kp42 | rectal swab | + | KPC | 2 |
| Kp43 | rectal swab | + | KPC | 16 |
| Kp44 | rectal swab | + | KPC | 2 |
| Kp45 | rectal swab | + | KPC | 32 |
| Kp46 | rectal swab | + | KPC | 4 |
| Kp47 | rectal swab | + | KPC | 32 |
| Kp48 | rectal swab | + | KPC | 8 |
| Kp49 | rectal swab | + | OXA-48 | 1 |
| Kp50 | rectal swab | + | KPC | 16 |
| Kp51 | rectal swab | + | KPC | 32 |
| Kp52 | rectal swab | + | KPC | 2 |
| Kp53 | biopsy | + | KPC | 64 |
| Kp54 | rectal swab | + | KPC | 32 |
| Kp55 | rectal swab | + | KPC | 32 |
| Kp56 | blood | + | KPC | 16 |
| Kp57 | urine | - | wt | 0.03 |
| Kp58 | urine | - | ESBL | 0.03 |
| Kp59 | urine | - | wt | 0.03 |
| Kp60 | urine | - | ESBL | 0.125 |
| Kp61 | urine | - | wt | 0.06 |
| Kp62 | urine | - | wt | 0.03 |
| Kp63 | urine | - | wt | 0.015 |

**S1 Table.**
