# Supplementary material for: Impact of meropenem on Klebsiella pneumoniae metabolism
Source: PLoS One. 2018 Nov 15;13(11):e0207478. doi: 10.1371/journal.pone.0207478 (PMC6237392; doi:10.1371/journal.pone.0207478)
Supplement: S2 Table — (DOCX) [file pone.0207478.s004.docx]

| **Intracellular metabolome** | **Extracellular metabolome** |
| --- | --- |
| Formate | Formate |
| Uridine | Histamine |
| Lactate | Tryptophan |
| Glycerol | Phenylalanine |
| Glycine | Tyrosine |
| Methanol | 4-Hydroxyphenylacetate |
| Betaine | 5-Hydroxytryptophan |
| Choline | Fumarate |
| Malonate | Urocanate |
| Homocitrulline | Uracil |
| Lysine | Citraconate |
| Succinate | Maltose |
| Pyruvate | Lactate |
| Glutamate | myo-Inositol |
| Acetone | Serine |
| Acetate | 4-Hydroxy-3-methoxymandelate |
| Alanine | Threonine |
| Ethanol | Glycine |
| Isobutyrate | Proline |
| Isocaproate | Agmatine |
|  | sn-Glycero-3-phosphocholine |
|  | Choline |
|  | Lysine |
|  | Cadaverine |
|  | Aspartate |
|  | Sarcosine |
|  | Methionine |
|  | Pyroglutamate |
|  | Succinate |
|  | Pyruvate |
|  | Glutamate |
|  | Acetone |
|  | Acetate |
|  | Alanine |
|  | 2-Hydroxyisobutyrate |
|  | Ethanol |
|  | Propionate |
|  | Isoleucine |
|  | Valine |
|  | Leucine |

**S2 Table.**
