# Supplementary material for: Impact of meropenem on Klebsiella pneumoniae metabolism
Source: PLoS One. 2018 Nov 15;13(11):e0207478. doi: 10.1371/journal.pone.0207478 (PMC6237392; doi:10.1371/journal.pone.0207478)
Supplement: S3 Table — The concentration of metabolites is expressed as mmol/L (mean ± SD). (DOCX) [file pone.0207478.s005.docx]

| **Internal metabolites** | **No stress** | **Meropenem stress** | **variation** | ***P*** |
| --- | --- | --- | --- | --- |
| **Overflow metabolites & organic acids** |  |  |  |  |
| Formate | 0.067 ± 0.04 | 0.021 ± 0.003 | ↓ | 0.008 |
| Acetate | 0.138 ± 0.05 | 0.023 ± 0.002 | ↓ | 0.008 |
| Isobutyrate | 0.004 ± 0.001 | 0.0007 ± 0.0003 | ↓ | 0.008 |
| Succinate | 0.014 ± 0.005 | 0.024 ± 0.005 | ↑ | 0.01 |
| Acetone | 0.002 ± 0.0007 | 0.001 ± 0.0004 | ↓ | 0.01 |
| Isocaproate | 0.008 ± 0.001 | 0.0009 ± 0.0006 | ↓ | 0.008 |
| Glycerol | 0.014 ± 0.003 | 0.006 ± 0.001 | ↓ | 0.008 |
| **Amino acids** |  |  |  |  |
| Glycine | 0.003 ± 0.001 | 0.001 ± 0.0006 | ↓ | 0.008 |
| Betaine | 0.006 ± 0.006 | 0.001 ± 0.001 | ↓ | 0.02 |

**S3 Table.**
