# Supplementary material for: Impact of meropenem on Klebsiella pneumoniae metabolism
Source: PLoS One. 2018 Nov 15;13(11):e0207478. doi: 10.1371/journal.pone.0207478 (PMC6237392; doi:10.1371/journal.pone.0207478)
Supplement: S4 Table — The concentration of metabolites is expressed as mmol/L (mean ± SD). (DOCX) [file pone.0207478.s006.docx]

| **External metabolites** | **No stress** | **Meropenem stress** | **variation** | ***P*** |
| --- | --- | --- | --- | --- |
| **Overflow metabolites & organic acids** |  |  |  |  |
| Formate | 3.741 ± 3.20 | 0.100 ± 0.09 | ↓ | 0.008 |
| Urocanate | 0.022 ± 0.011 | 0.005 ± 0.004 | ↓ | 0.008 |
| Citraconate | 0.018 ± 0.001 | 0.014 ± 0.001 | ↓ | 0.014 |
| Lactate | 0.689 ± 0.207 | -0.040 ± 0.006 | ↓ | 0.008 |
| 4-H-3-methoxymandelate | 0.07 ± 0.007 | 0.058 ± 0.005 | ↓ | 0.015 |
| Pyroglutamate | 1.189 ± 0.07 | 1.116 ± 0.05 | ↓ | 0.03 |
| Succinate | 4.923 ± 0.995 | 1.846 ± 0.180 | ↓ | 0.008 |
| Pyruvate | 0.283 ± 0.514 | 1.026 ± 0.150 | ↑ | 0.01 |
| Acetone | 0.051 ± 0.005 | 0.056 ± 0.002 | ↑ | 0.04 |
| Acetate | 14.46 ± 1.414 | 7.699 ± 1.399 | ↓ | 0.008 |
| 2-Hydroxyisobutyrate | 0.046 ± 0.018 | 0.192 ± 0.006 | ↑ | 0.008 |
| Ethanol | 0.518 ± 0.686 | 0.176 ± 0.06 | ↓ | 0.008 |
| Propionate | 1.581 ± 0.321 | 0.132 ± 0.221 | ↓ | 0.008 |
| **Amino acids** |  |  |  |  |
| Serine | 0.604 ± 0.595 | 1.976 ± 0.431 | ↑ | 0.015 |
| Alanine | 3.966 ± 0.359 | 3.203 ± 0.08 | ↓ | 0.008 |
| Isoleucine | 1.076 ± 0.08 | 0.960 ± 0.04 | ↓ | 0.04 |
| Aspartate | 0.537 ± 0.519 | 1.760 ± 0.120 | ↑ | 0.008 |
| Threonine | 0.275 ± 0.445 | 2.300 ± 0.543 | ↑ | 0.008 |
| Lysine | 0.639 ± 0.668 | 2.110 ± 0.154 | ↑ | 0.008 |
| Glutamate | 12.79 ± 0.379 | 12.38 ± 0.205 | ↓ | 0.01 |
| Cadaverine | 2.589 ± 0.735 | 1.062 ± 0.231 | ↓ | 0.008 |
| **Others** |  |  |  |  |
| Maltose | 0.020 ± 0.007 | 0.006 ± 0.001 | ↓ | 0.008 |

**S4 Table.**
